# (*Z*)-3-Meth­oxy-*N*-[(5-nitro­thio­phen-2-yl)methyl­idene]aniline

**DOI:** 10.1107/S1600536812033120

**Published:** 2012-08-11

**Authors:** Tufan Akbal, Erbil Ağar, Ahmet Erdönmez

**Affiliations:** a Ondokuz Mayıs University, Arts and Sciences Faculty, Department of Physics, 55139 Samsun, Turkey; b Ondokuz Mayıs University, Arts and Sciences Faculty, Department of Chemistry, 55139 Samsun, Turkey

## Abstract

In the title compound, C_12_H_10_N_2_O_3_S, the dihedral angle between the benzene and thio­phene rings is 43.17 (4)°. The crystal structure is devoid of any hydrogen-bonding inter­actions. However, π–π inter­actions between the benzene and thio­phene rings [distance between ring centroids = 3.6850 (11) Å] stack the mol­ecules along the *a* axis. The absolute structure could not be determined as the crystal studied was a racemic twin with a BASF parameter of 0.31 (6).

## Related literature

For biological and industrial properties of Schiff bases, see: Barton & Ollis (1979 ▶); Taggi *et al.* (2002 ▶). For a related structure, see: Ceylan *et al.* (2011 ▶).
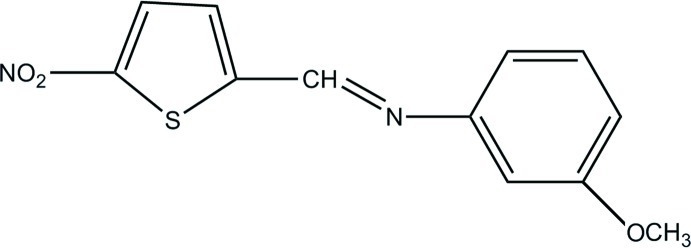


## Experimental

### 

#### Crystal data

C_12_H_10_N_2_O_3_S
*M* *_r_* = 262.29
Orthorhombic, 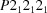

*a* = 7.4612 (3) Å
*b* = 10.8737 (5) Å
*c* = 14.8465 (9) Å
*V* = 1204.51 (10) Å^3^
*Z* = 4
Mo *K*α radiation
μ = 0.27 mm^−1^
*T* = 296 K
0.69 × 0.51 × 0.28 mm

#### Data collection

Stoe IPDS 2 diffractometer
Absorption correction: integration (*X-RED*; Stoe & Cie, 2002 ▶) *T*_min_ = 0.873, *T*_max_ = 0.938
5538 measured reflections
2375 independent reflections
2200 reflections with *I* > 2σ(*I*)
*R*_int_ = 0.034

#### Refinement

*R*[*F* ^2^ > 2σ(*F* ^2^)] = 0.030
*wR*(*F* ^2^) = 0.076
*S* = 1.02
2375 reflections
165 parameters
H-atom parameters constrained
Δρ_max_ = 0.17 e Å^−3^
Δρ_min_ = −0.18 e Å^−3^
Absolute structure: Flack (1983 ▶), 986 Friedel pairs
Flack parameter: 0.31 (6)

### 

Data collection: *X-AREA* (Stoe & Cie, 2002 ▶); cell refinement: *X-AREA*; data reduction: *X-RED32* (Stoe & Cie, 2002 ▶); program(s) used to solve structure: *SHELXS97* (Sheldrick, 2008 ▶); program(s) used to refine structure: *SHELXL97* (Sheldrick, 2008 ▶); molecular graphics: *ORTEP-3 for Windows* (Farrugia, 1997 ▶); software used to prepare material for publication: *WinGX* (Farrugia, 1999 ▶) and *PLATON* (Spek, 2009 ▶).

## Supplementary Material

Crystal structure: contains datablock(s) I, global. DOI: 10.1107/S1600536812033120/pv2561sup1.cif

Structure factors: contains datablock(s) I. DOI: 10.1107/S1600536812033120/pv2561Isup2.hkl

Supplementary material file. DOI: 10.1107/S1600536812033120/pv2561Isup4.mol

Supplementary material file. DOI: 10.1107/S1600536812033120/pv2561Isup4.cml

Additional supplementary materials:  crystallographic information; 3D view; checkCIF report

## Figures and Tables

**Fig. 1.**
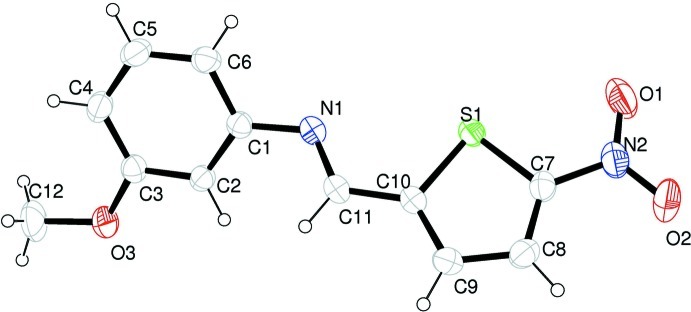
The molecular structure of title molecule with the atom-numbering scheme. Displacement ellipsoids are drawn at the 30% probability level.

**Fig. 2.**
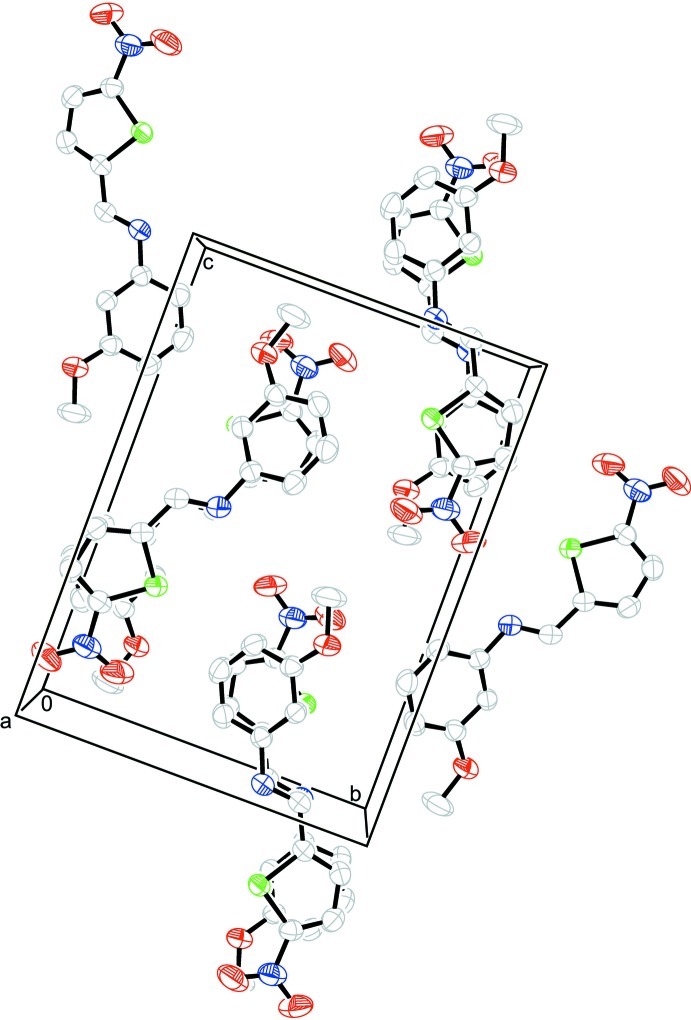
A view of the crystal packing of the title compound. Hydrogen atoms have been excluded for clarity.

## supplementary crystallographic information

### Comment

Schiff bases are used as starting materials in the synthesis of important drugs, such as antibiotics and antiallergic, antiphlogistic, and antitumor substances (Barton & Ollis, 1979). In addition, they have a wide range of industrial applications, such as dyes and pigments (Taggi *et al.*, 2002). Herein we report the synthesis and crystal structure of the title compound.

In the title compound (Fig. 1), the dihedral angle between the nitro-thiophene (C7—C10/S1) and the benzene ring (Cl—C6) is 47.14 (4) °. The bond distances and angles in the title compound agree very well with the corresponding bond distances and angles reported in a closely related compound (Ceylan *et al.* 2011). The structure is devoid of any hydrogen bonding interactions. However, π—π interactions between the centroids of the benzene and thiophene rings (distance between ring centroids = 3.6850 (11) Å) are observed in the crystal structure.

### Experimental

The compound title compound was prepared by refluxing a mixture of a solution of 5-nitro-2-thiophene-carboxaldehyde (0.018 g, 0.120 mmol) in ethanol (20 ml) and a solution of 3-methoxyaniline (0.0142 g, 0.120 mmol) in ethanol (20 ml). The reaction mixture was stirred for 1 h under reflux. The crystals of the title compound suitable for X-ray analysis were obtained from ethanol by slow evaporation (yield %61; m.p 385–386 K).

### Refinement

All H atoms were positioned geometrically and refined using a riding model, with C—H = 0.93 and 0.96 Å, for aryl and methyl H-atoms, respectively. The *U*_iso_(H) were allowed at 1.5*U*_eq_(C methyl) or 1.2*U*_eq_(C aryl). The compound crystallized as a racemic twin as indicated by *SHELXL97* (Sheldrick, 2008). A twin refinement using the commands TWIN and BASF gave a twin fraction of 0.31 (6)/0.69 (6); 986 Friedel pairs of reflections were not merged.

### Crystal data

**Table:**

C_12_H_10_N_2_O_3_S	*F*(000) = 544
*M**_r_* = 262.29	*D*_x_ = 1.446 Mg m^−^^3^
Orthorhombic, *P*2_1_2_1_2_1_	Mo *K*α radiation, λ = 0.71073 Å
Hall symbol: P 2ac 2ab	Cell parameters from 5538 reflections
*a* = 7.4612 (3) Å	θ = 2.3–26°
*b* = 10.8737 (5) Å	µ = 0.27 mm^−^^1^
*c* = 14.8465 (9) Å	*T* = 296 K
*V* = 1204.51 (10) Å^3^	Prism, yellow
*Z* = 4	0.69 × 0.51 × 0.28 mm

### Data collection

**Table:**

Stoe IPDS 2 diffractometer	2375 independent reflections
Radiation source: fine-focus sealed tube	2200 reflections with *I* > 2σ(*I*)
Graphite monochromator	*R*_int_ = 0.034
w–scan rotation	θ_max_ = 26.0°, θ_min_ = 2.3°
Absorption correction: integration (*X-RED*; Stoe & Cie, 2002)	*h* = −8→9
*T*_min_ = 0.873, *T*_max_ = 0.938	*k* = −13→13
5538 measured reflections	*l* = −15→18

### Refinement

**Table:**

Refinement on *F*^2^	Hydrogen site location: inferred from neighbouring sites
Least-squares matrix: full	H-atom parameters constrained
*R*[*F*^2^ > 2σ(*F*^2^)] = 0.030	*w* = 1/[σ^2^(*F*_o_^2^) + (0.0517*P*)^2^] where *P* = (*F*_o_^2^ + 2*F*_c_^2^)/3
*wR*(*F*^2^) = 0.076	(Δ/σ)_max_ = 0.001
*S* = 1.02	Δρ_max_ = 0.17 e Å^−^^3^
2375 reflections	Δρ_min_ = −0.18 e Å^−^^3^
165 parameters	Extinction correction: *SHELXL97* (Sheldrick, 2008), Fc^*^=kFc[1+0.001xFc^2^λ^3^/sin(2θ)]^-1/4^
0 restraints	Extinction coefficient: 0.0097 (18)
Primary atom site location: structure-invariant direct methods	Absolute structure: Flack (1983),986 Friedel pairs
Secondary atom site location: difference Fourier map	Flack parameter: 0.31 (6)

### Special details

**Table:**

Geometry. All e.s.d.'s (except the e.s.d. in the dihedral angle between two l.s. planes) are estimated using the full covariance matrix. The cell e.s.d.'s are taken into account individually in the estimation of e.s.d.'s in distances, angles and torsion angles; correlations between e.s.d.'s in cell parameters are only used when they are defined by crystal symmetry. An approximate (isotropic) treatment of cell e.s.d.'s is used for estimating e.s.d.'s involving l.s. planes.
Refinement. Refinement of *F*^2^ against ALL reflections. The weighted *R*-factor *wR* and goodness of fit *S* are based on *F*^2^, conventional *R*-factors *R* are based on *F*, with *F* set to zero for negative *F*^2^. The threshold expression of *F*^2^ > σ(*F*^2^) is used only for calculating *R*-factors(gt) *etc*. and is not relevant to the choice of reflections for refinement. *R*-factors based on *F*^2^ are statistically about twice as large as those based on *F*, and *R*- factors based on ALL data will be even larger.

### Fractional atomic coordinates and isotropic or equivalent isotropic displacement parameters (Å^2^)

**Table:**

	*x*	*y*	*z*	*U*_iso_*/*U*_eq_	
S1	0.17537 (6)	0.72680 (4)	0.17461 (3)	0.04740 (13)	
O3	0.2561 (2)	0.78260 (13)	−0.33068 (9)	0.0645 (3)	
N1	0.1374 (2)	0.81303 (13)	−0.01636 (9)	0.0475 (3)	
N2	0.1792 (2)	0.60779 (17)	0.33474 (11)	0.0665 (4)	
O1	0.2270 (3)	0.70702 (19)	0.36455 (10)	0.0961 (6)	
C1	0.1284 (2)	0.86180 (14)	−0.10418 (11)	0.0440 (4)	
C3	0.1854 (2)	0.85202 (15)	−0.26249 (11)	0.0479 (4)	
C7	0.1414 (2)	0.60021 (16)	0.24090 (12)	0.0490 (4)	
C10	0.1066 (2)	0.64354 (15)	0.08297 (11)	0.0449 (4)	
C4	0.1172 (3)	0.96946 (18)	−0.27339 (13)	0.0585 (5)	
H4	0.1131	1.0056	−0.3301	0.070*	
C6	0.0617 (3)	0.98144 (16)	−0.11459 (13)	0.0539 (4)	
H6	0.0224	1.0258	−0.0648	0.065*	
O2	0.1619 (3)	0.51571 (17)	0.38055 (10)	0.0973 (6)	
C8	0.0809 (3)	0.50020 (17)	0.19685 (13)	0.0560 (5)	
H8	0.0555	0.4250	0.2238	0.067*	
C5	0.0552 (3)	1.03209 (18)	−0.19860 (14)	0.0619 (5)	
H5	0.0079	1.1106	−0.2057	0.074*	
C2	0.1906 (2)	0.79816 (13)	−0.17871 (11)	0.0442 (3)	
H2	0.2360	0.7190	−0.1721	0.053*	
C11	0.0970 (2)	0.70084 (16)	−0.00462 (11)	0.0471 (4)	
H11	0.0602	0.6542	−0.0538	0.057*	
C9	0.0616 (3)	0.52531 (17)	0.10520 (13)	0.0546 (4)	
H9	0.0222	0.4676	0.0634	0.065*	
C12	0.2302 (4)	0.8228 (3)	−0.42063 (14)	0.0905 (8)	
H12A	0.2864	0.7659	−0.4612	0.136*	
H12B	0.2824	0.9028	−0.4281	0.136*	
H12C	0.1042	0.8268	−0.4334	0.136*	

### Atomic displacement parameters (Å^2^)

**Table:**

	*U*^11^	*U*^22^	*U*^33^	*U*^12^	*U*^13^	*U*^23^
S1	0.0552 (2)	0.0474 (2)	0.03953 (19)	−0.00386 (17)	0.00184 (18)	−0.00330 (17)
O3	0.0855 (9)	0.0682 (8)	0.0397 (6)	0.0033 (7)	0.0063 (6)	0.0038 (6)
N1	0.0494 (7)	0.0529 (7)	0.0401 (7)	−0.0007 (6)	−0.0015 (6)	−0.0038 (6)
N2	0.0752 (11)	0.0795 (10)	0.0447 (8)	0.0042 (9)	0.0000 (9)	0.0045 (8)
O1	0.1360 (17)	0.1054 (13)	0.0468 (8)	−0.0233 (12)	−0.0092 (9)	−0.0107 (9)
C1	0.0418 (8)	0.0459 (8)	0.0444 (9)	−0.0028 (7)	−0.0040 (7)	−0.0006 (7)
C3	0.0481 (9)	0.0512 (8)	0.0443 (8)	−0.0065 (8)	−0.0015 (8)	0.0033 (7)
C7	0.0494 (9)	0.0555 (9)	0.0421 (8)	0.0057 (8)	0.0031 (7)	0.0042 (7)
C10	0.0449 (9)	0.0496 (8)	0.0403 (8)	0.0003 (7)	0.0005 (7)	−0.0045 (7)
C4	0.0659 (11)	0.0538 (9)	0.0558 (10)	−0.0045 (9)	−0.0125 (9)	0.0135 (9)
C6	0.0565 (10)	0.0463 (9)	0.0590 (11)	0.0008 (8)	−0.0086 (9)	−0.0075 (8)
O2	0.1395 (16)	0.0977 (11)	0.0546 (9)	0.0113 (13)	0.0039 (10)	0.0283 (9)
C8	0.0616 (11)	0.0467 (9)	0.0598 (11)	0.0012 (7)	0.0033 (9)	0.0068 (8)
C5	0.0670 (11)	0.0445 (9)	0.0742 (13)	0.0061 (8)	−0.0134 (10)	0.0034 (9)
C2	0.0451 (8)	0.0418 (7)	0.0456 (8)	−0.0014 (6)	−0.0029 (7)	0.0026 (7)
C11	0.0485 (9)	0.0546 (9)	0.0382 (8)	−0.0010 (7)	−0.0002 (7)	−0.0056 (7)
C9	0.0621 (10)	0.0482 (9)	0.0534 (10)	0.0002 (8)	−0.0032 (9)	−0.0078 (8)
C12	0.118 (2)	0.1166 (19)	0.0373 (10)	0.0071 (17)	0.0024 (12)	0.0116 (12)

### Geometric parameters (Å, º)

**Table:**

S1—C7	1.7110 (18)	C10—C11	1.444 (2)
S1—C10	1.7128 (17)	C4—C5	1.382 (3)
O3—C3	1.369 (2)	C4—H4	0.9300
O3—C12	1.418 (3)	C6—C5	1.364 (3)
N1—C11	1.269 (2)	C6—H6	0.9300
N1—C1	1.409 (2)	C8—C9	1.395 (3)
N2—O2	1.217 (2)	C8—H8	0.9300
N2—O1	1.220 (2)	C5—H5	0.9300
N2—C7	1.424 (2)	C2—H2	0.9300
C1—C2	1.385 (2)	C11—H11	0.9300
C1—C6	1.401 (2)	C9—H9	0.9300
C3—C2	1.375 (2)	C12—H12A	0.9600
C3—C4	1.384 (3)	C12—H12B	0.9600
C7—C8	1.347 (3)	C12—H12C	0.9600
C10—C9	1.369 (3)		
			
C7—S1—C10	89.27 (8)	C1—C6—H6	120.4
C3—O3—C12	118.30 (17)	C7—C8—C9	110.49 (17)
C11—N1—C1	118.54 (14)	C7—C8—H8	124.8
O2—N2—O1	123.77 (18)	C9—C8—H8	124.8
O2—N2—C7	118.57 (18)	C6—C5—C4	121.58 (17)
O1—N2—C7	117.66 (17)	C6—C5—H5	119.2
C2—C1—C6	119.65 (16)	C4—C5—H5	119.2
C2—C1—N1	122.36 (14)	C3—C2—C1	120.01 (14)
C6—C1—N1	117.92 (15)	C3—C2—H2	120.0
O3—C3—C2	115.04 (15)	C1—C2—H2	120.0
O3—C3—C4	124.34 (16)	N1—C11—C10	121.81 (15)
C2—C3—C4	120.60 (16)	N1—C11—H11	119.1
C8—C7—N2	126.02 (17)	C10—C11—H11	119.1
C8—C7—S1	114.83 (13)	C10—C9—C8	113.18 (17)
N2—C7—S1	119.15 (14)	C10—C9—H9	123.4
C9—C10—C11	127.61 (16)	C8—C9—H9	123.4
C9—C10—S1	112.23 (14)	O3—C12—H12A	109.5
C11—C10—S1	120.14 (12)	O3—C12—H12B	109.5
C5—C4—C3	118.92 (17)	H12A—C12—H12B	109.5
C5—C4—H4	120.5	O3—C12—H12C	109.5
C3—C4—H4	120.5	H12A—C12—H12C	109.5
C5—C6—C1	119.22 (18)	H12B—C12—H12C	109.5
C5—C6—H6	120.4		
			
C11—N1—C1—C2	−42.6 (2)	N1—C1—C6—C5	178.55 (16)
C11—N1—C1—C6	140.62 (16)	N2—C7—C8—C9	−179.03 (19)
C12—O3—C3—C2	170.13 (19)	S1—C7—C8—C9	0.5 (2)
C12—O3—C3—C4	−11.7 (3)	C1—C6—C5—C4	−1.7 (3)
O2—N2—C7—C8	2.6 (3)	C3—C4—C5—C6	0.8 (3)
O1—N2—C7—C8	−177.3 (2)	O3—C3—C2—C1	177.79 (15)
O2—N2—C7—S1	−176.88 (17)	C4—C3—C2—C1	−0.4 (3)
O1—N2—C7—S1	3.2 (3)	C6—C1—C2—C3	−0.6 (2)
C10—S1—C7—C8	−0.21 (15)	N1—C1—C2—C3	−177.34 (15)
C10—S1—C7—N2	179.32 (16)	C1—N1—C11—C10	178.65 (15)
C7—S1—C10—C9	−0.11 (15)	C9—C10—C11—N1	177.72 (17)
C7—S1—C10—C11	178.33 (15)	S1—C10—C11—N1	−0.5 (2)
O3—C3—C4—C5	−177.69 (18)	C11—C10—C9—C8	−177.90 (17)
C2—C3—C4—C5	0.3 (3)	S1—C10—C9—C8	0.4 (2)
C2—C1—C6—C5	1.6 (3)	C7—C8—C9—C10	−0.5 (2)
